# Supplementary figures and images for: Discovery of novel hepatocyte eQTLs in African Americans
Source: PLoS Genet. 2020 Apr 20;16(4):e1008662. doi: 10.1371/journal.pgen.1008662 (PMC7192504; doi:10.1371/journal.pgen.1008662)

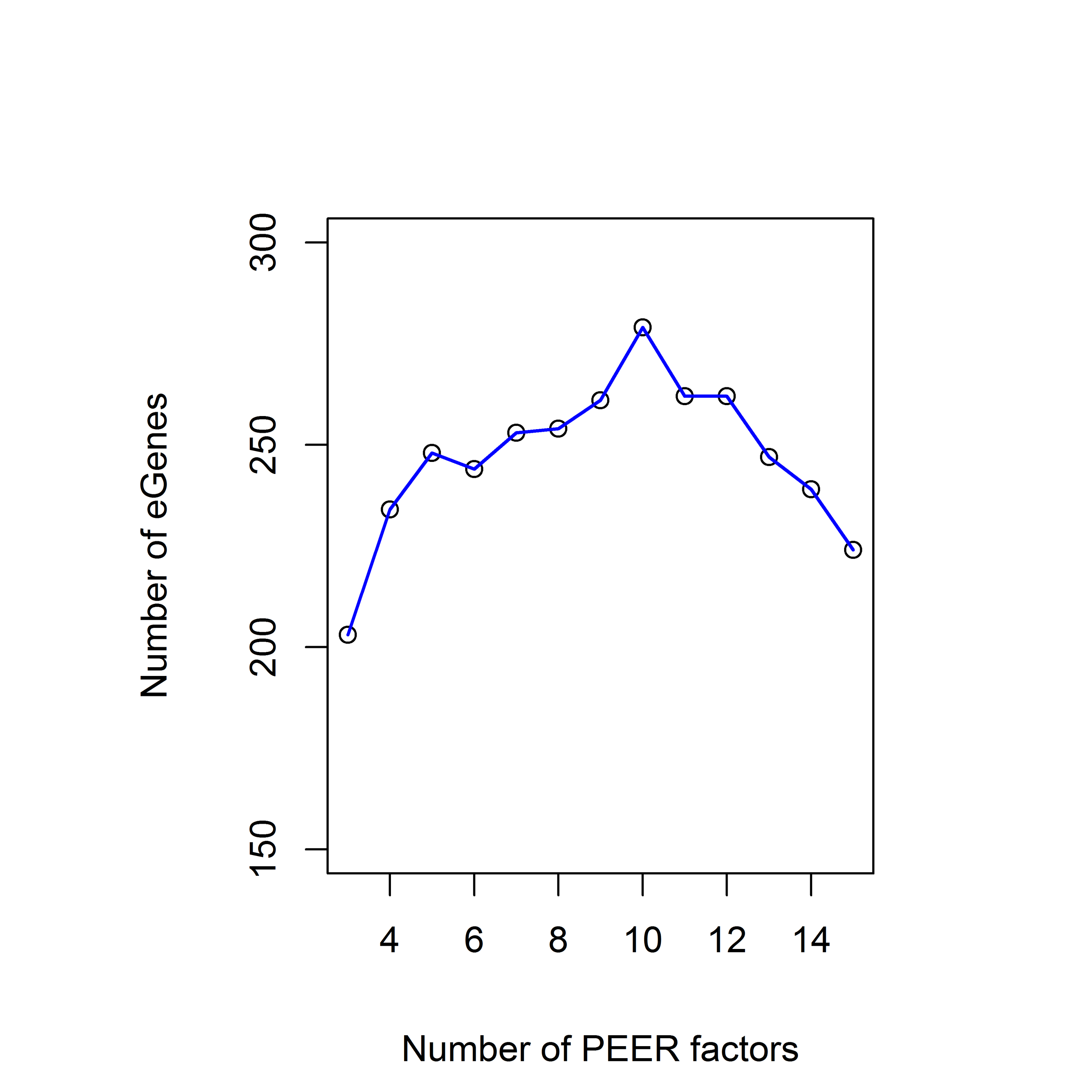

Supplement: S1 Fig — We tested different numbers of PEER variables as covariates in LA adjusted eQTL mapping of AA hepatocytes in order to maximize the power to discover eGenes. Using the hierarchical multi-testing correction method and the threshold of FDR<0.05, we found that 10 PEERs gave the largest number of discovered eGenes and this result was used in the downstream eQTL mapping. (TIF) [file pgen.1008662.s001.tif]

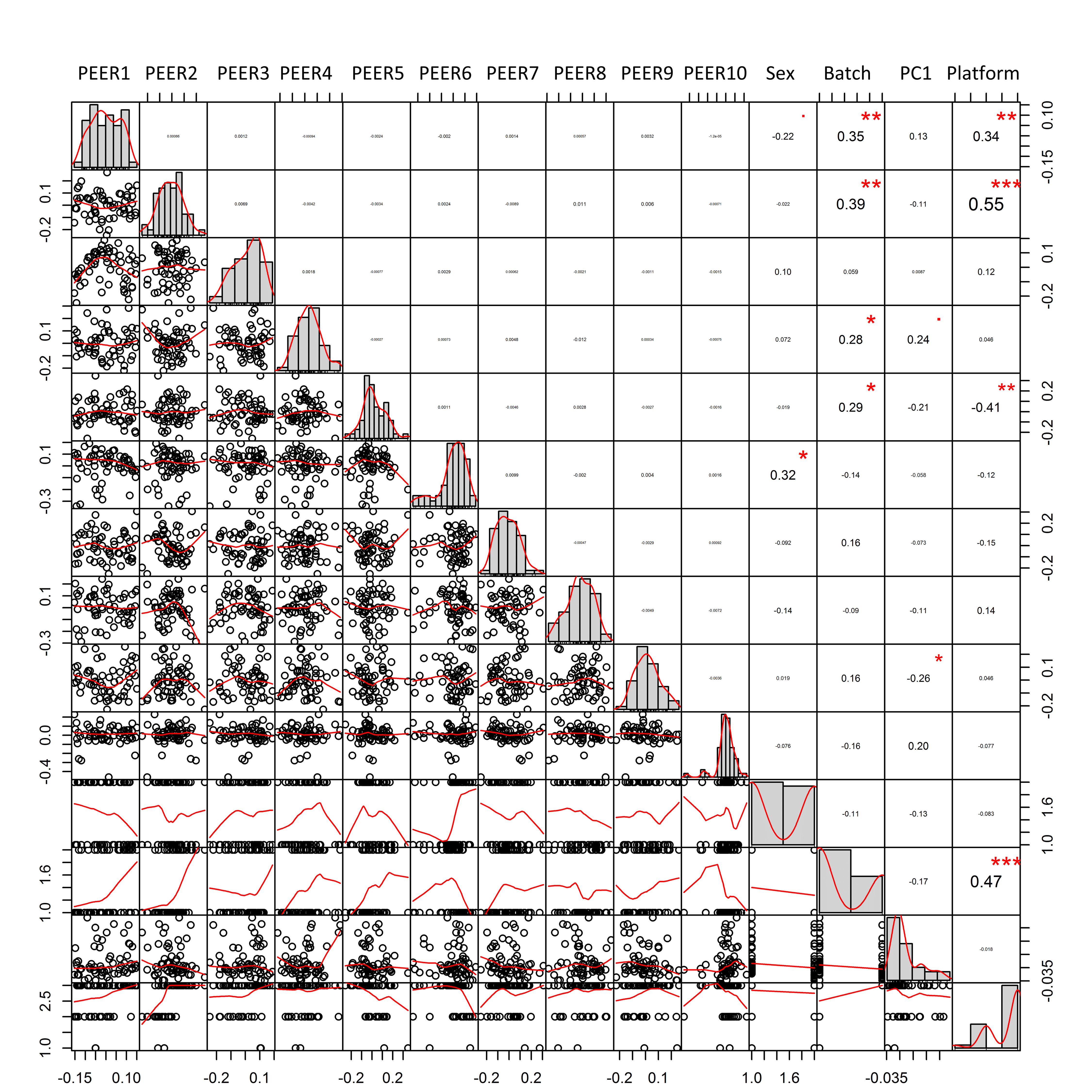

Supplement: S2 Fig — We plot the pair-wise correlation of the covariates used in the eQTL mapping model. The lower triangle shows the pair wise scatter plot with the red line representing the fitted regression line. The diagonal represents the histogram of each covariant. The upper triangle shows the Pearson correlation r for each pair of covariates with the significant correlation highlighted with red stars (p< 0.001 = ***, p<0.01 = **, p<0.05 = *, p<0.1 = dot). Due to the high correlation between PC1, PC2 and PC3 estimated from genotype data (also can be seen from S5 Fig), only PC1 was used as a covariate to account for population substructure. (TIF) [file pgen.1008662.s002.tif]

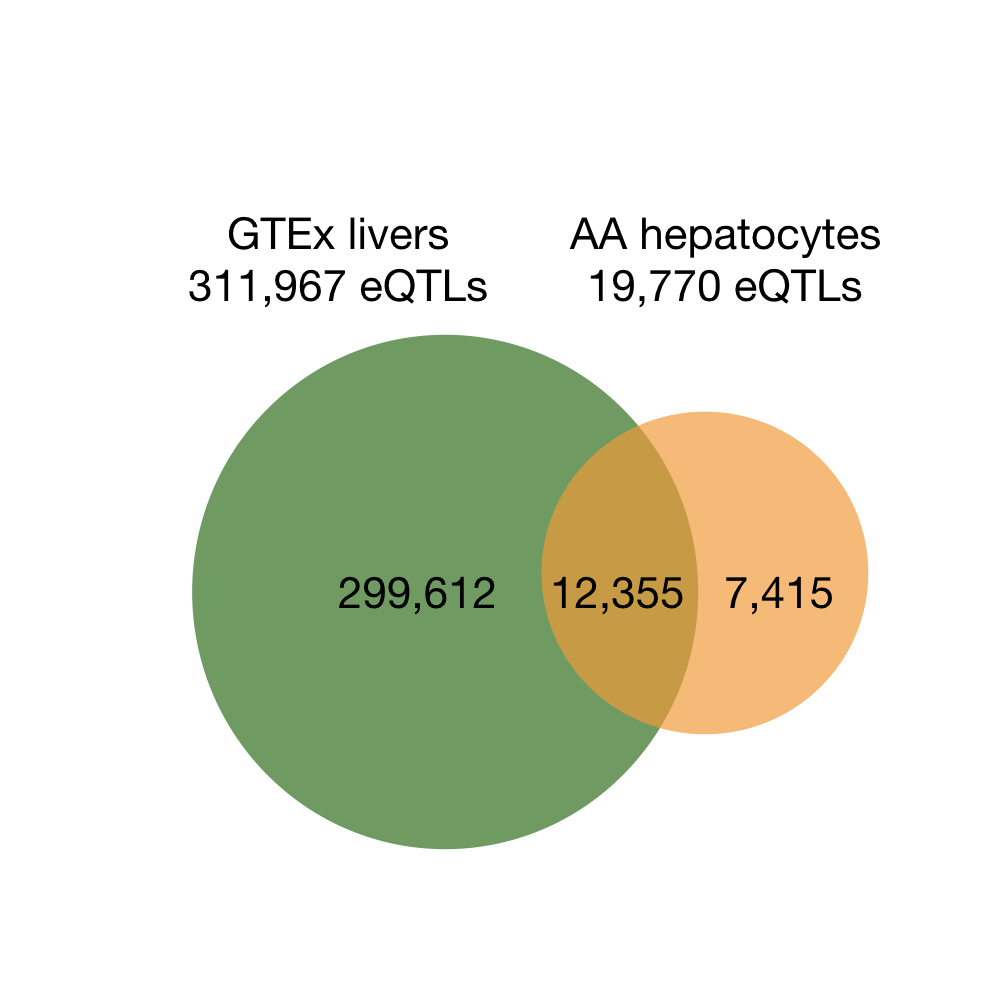

Supplement: S3 Fig — Approximately 62.50% (12,355) AA hepatocyte eQTLs were significant eQTLs within GTEx liver dataset and 37.51% (7,415) eQTLs were unique to the AA hepatocyte dataset. (TIF) [file pgen.1008662.s003.tif]

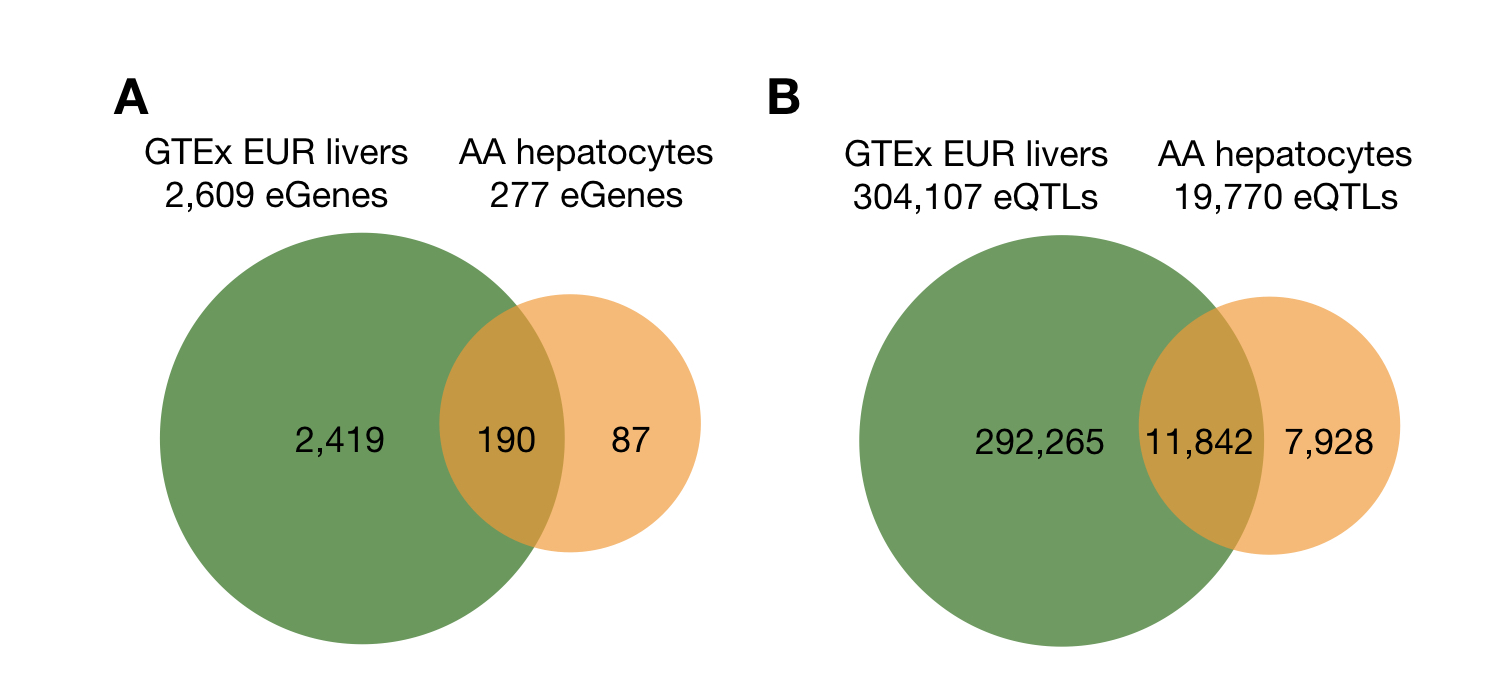

Supplement: S4 Fig — We selected 127 samples that are of European (EUR) ancestry in GTEx liver cohort (S5 Fig) and performed eQTL mapping with FastQTL. We did 1000 permutation to identify the threshold of eGene discovery and selected corresponding eQTLs at FDR<0.05. We found a similar pattern of overlapping as the main results present in the manuscript (Fig 1B and S3 Fig). (TIF) [file pgen.1008662.s004.tif]

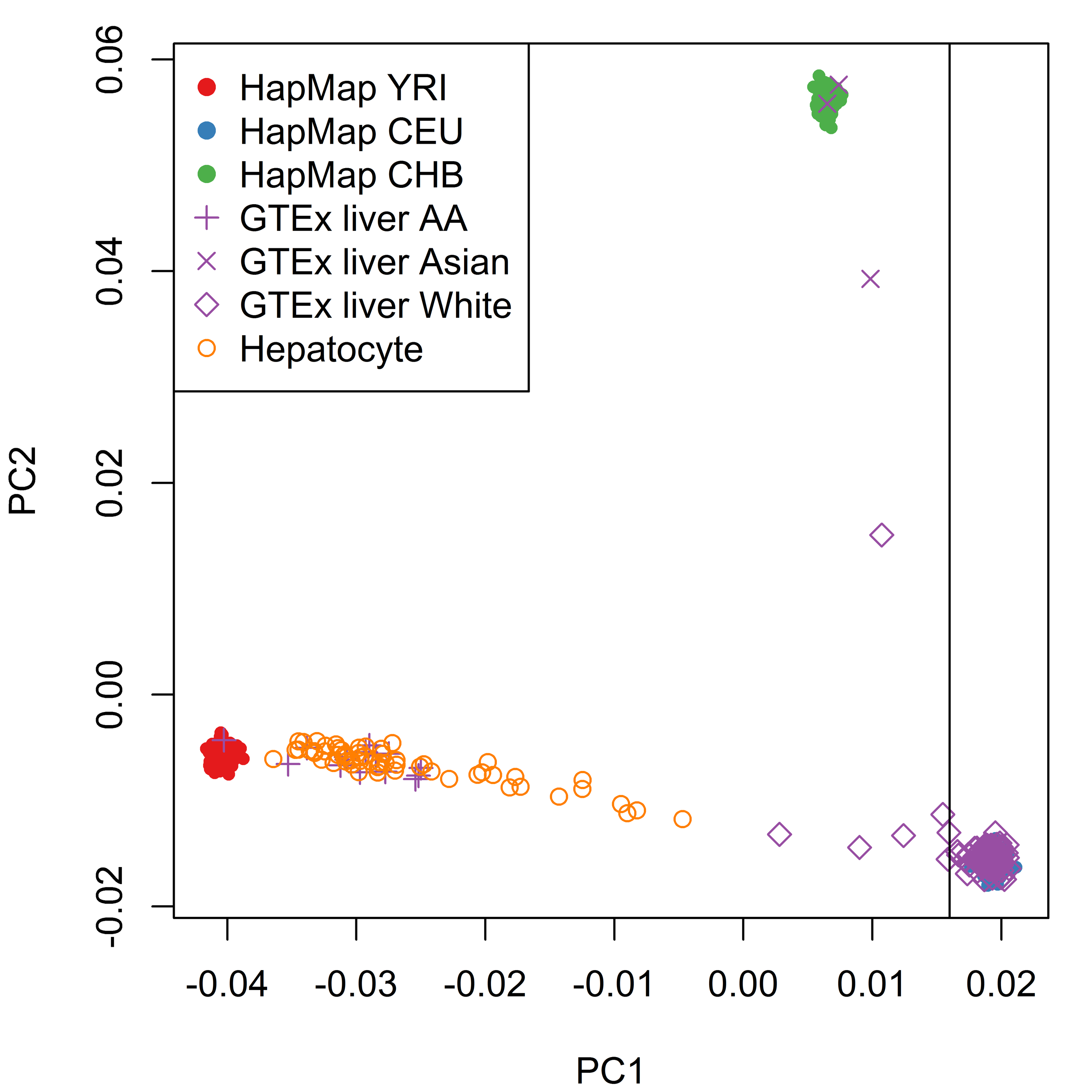

Supplement: S5 Fig — The GTEx liver samples are shown by the race/ethnicity provided in the GTEx phenotype file. The vertical line (PC1>0.016) was used to identify GTEx liver samples of European descent (n = 127). The GTEx AA samples (n = 15) and AA hepatocyte samples (n = 60) lie in the cluster between HapMap CEU and YRI. (TIF) [file pgen.1008662.s005.tif]

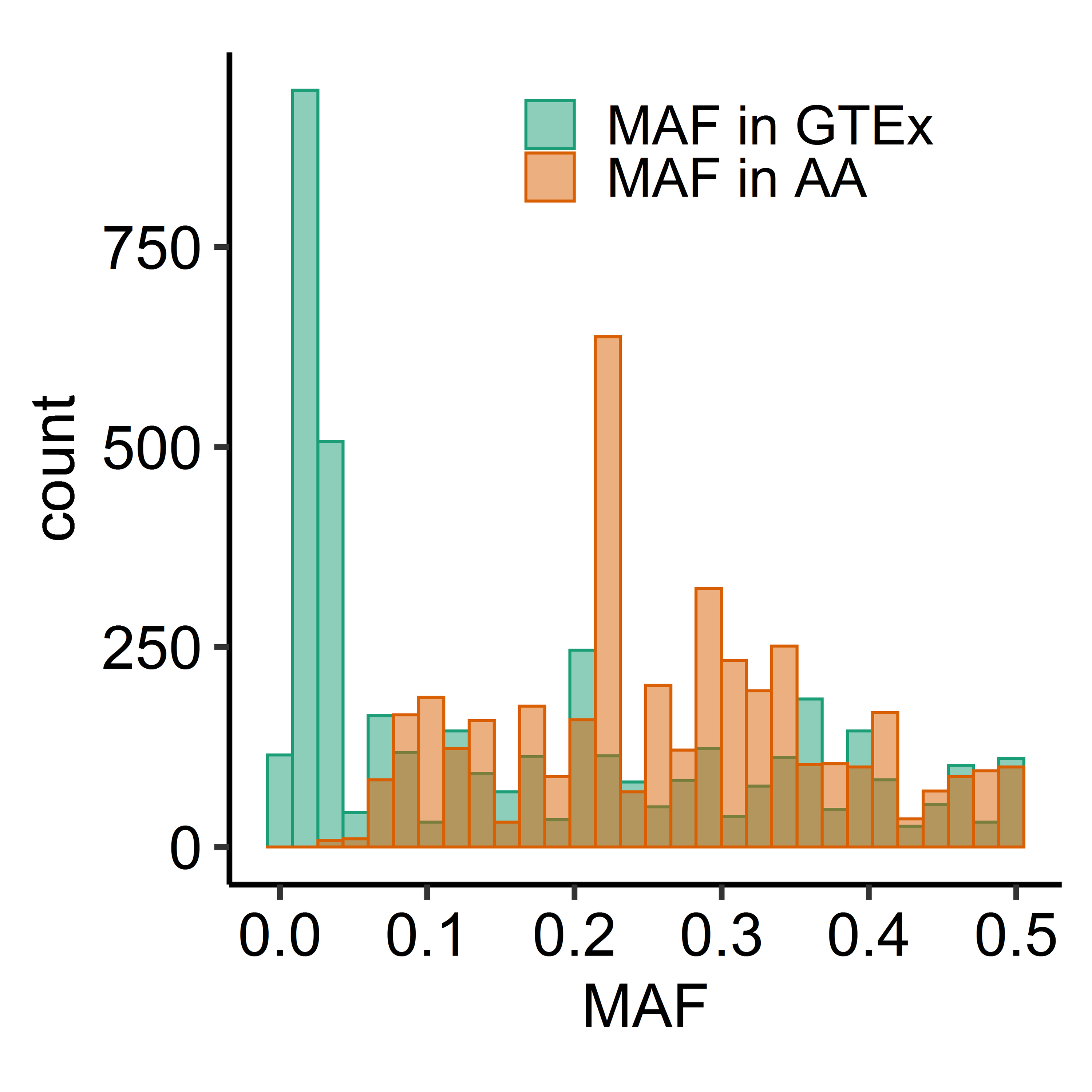

Supplement: S6 Fig — Among 7,415 AA-specific eQTLs, 4,291 were available in GTEx dataset. The comparison of the MAF (calculated in each cohort) show that AA-specific eQTLs are skewed towards small MAF in GTEx. Hence, the GTEx liver dataset may not have been powered to detect these SNPs as eQTLs. (TIF) [file pgen.1008662.s006.tif]

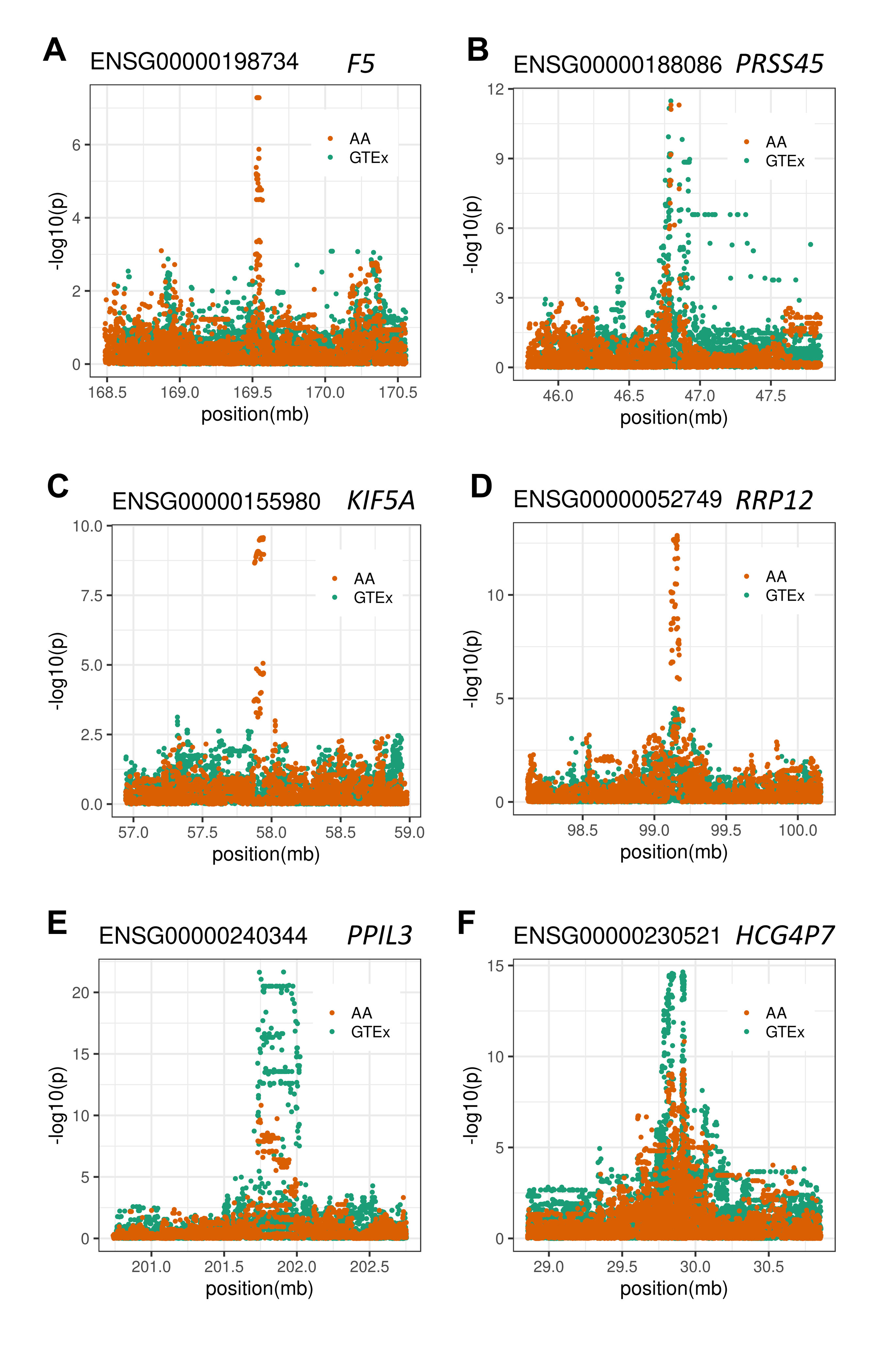

Supplement: S7 Fig — Regional plot for AA hepatocyte eGenes shows different patterns of associations. Plotted are the locusZoom plots showing the -log P values of cis SNPs to the labelled gene in either AA hepatocytes or GTEx livers. A) The unique eGene, F5, has associations with genetic variants specific to AA hepatocytes. B) In the PRSS45 gene, the AA hepatocyte eQTLs are in shorter stretches of LD than GTEx liver eQTLs. C) eQTLs for KIF5A are only found in AA hepatocyte dataset but not in GTEx liver dataset. D) The GWAS variant, rs7903847, which is associated with granulocyte percentage of myeloid white cells, is in LD with an AA-specific eQTL, rs10786336, for RRP12 gene. This SNP is not an eQTL in the GTEx liver analysis. E) and F) show the eGenes with secondary eQTLs. (TIF) [file pgen.1008662.s007.tif]

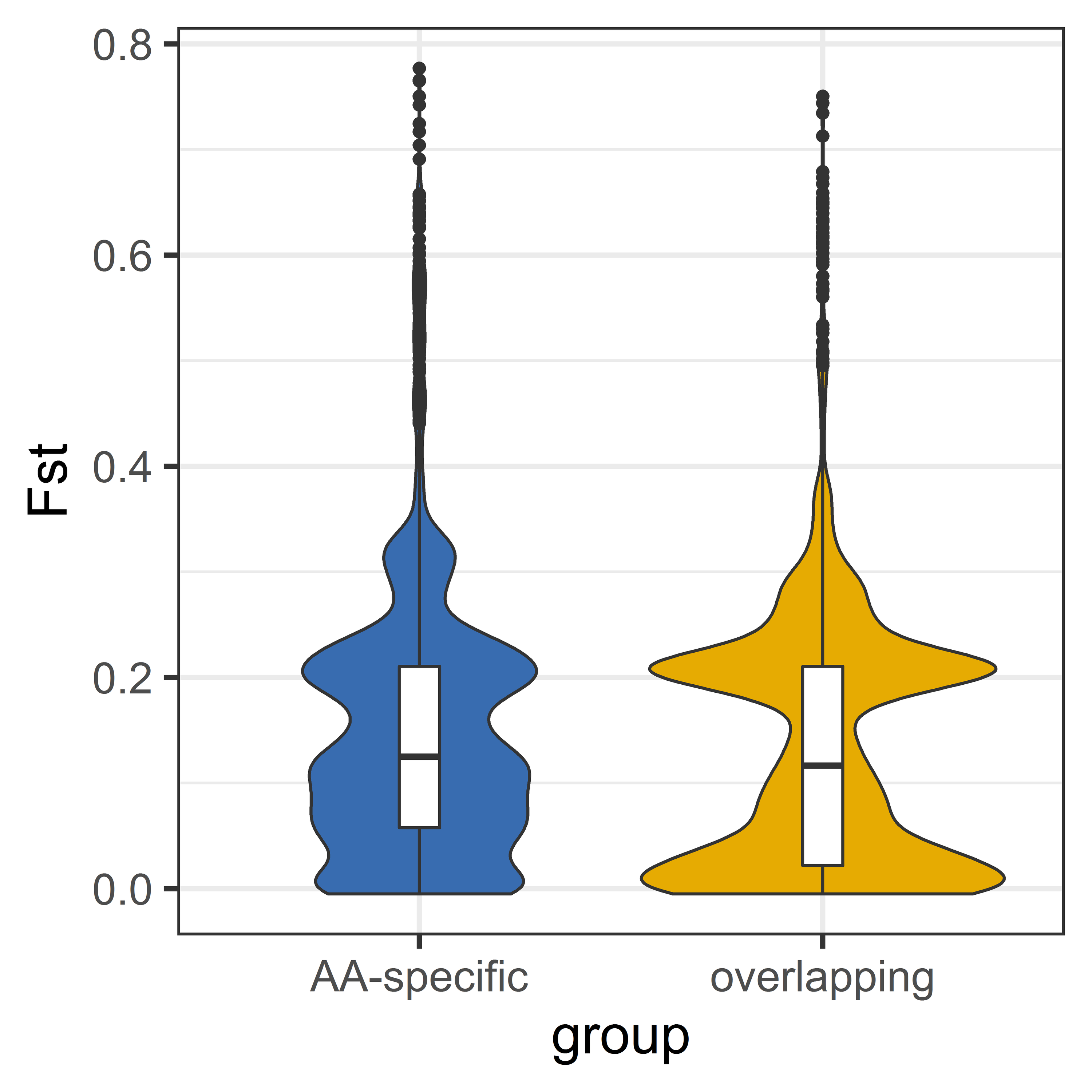

Supplement: S8 Fig — We calculated Fst values using 1000 Genome phase 3 data using YRI and CEU populations with GCTA. The Fst values for overlapping and AA-specific eQTLs were compared. Here shows that the AA-specific eQTLs have higher Fst than the overlapping eQTLs (Mann-Whitney U test, p = 1.82e-12, one-side). (TIF) [file pgen.1008662.s008.tif]

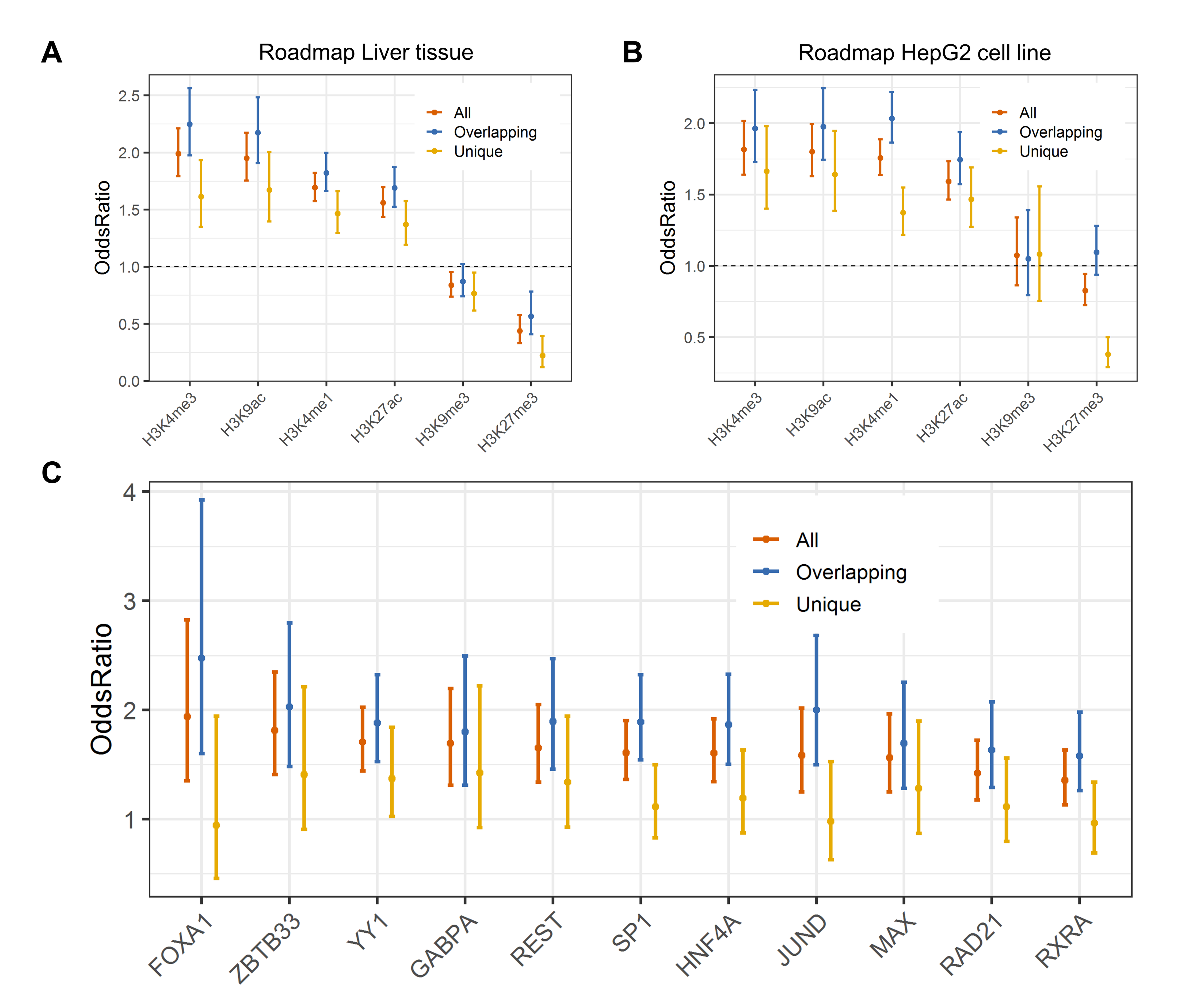

Supplement: S9 Fig — We tested the enrichment of AA hepatocyte eQTLs, overlapping eQTLs, and AA-specific eQTLs in Roadmap histone modifications mapped in liver tissue (A) and HepG2 cell line (B) compared with a matched null SNPs set. The H3K9me3 is no longer significantly depleted in HepG2, suggesting the different landscapes of histone modifications between liver tissue and HepG2 cell line. (C) shows the enrichment in TF binding in hepatocytes in ENCODE for all, overlapping and AA-specific eQTLs. (TIF) [file pgen.1008662.s009.tif]

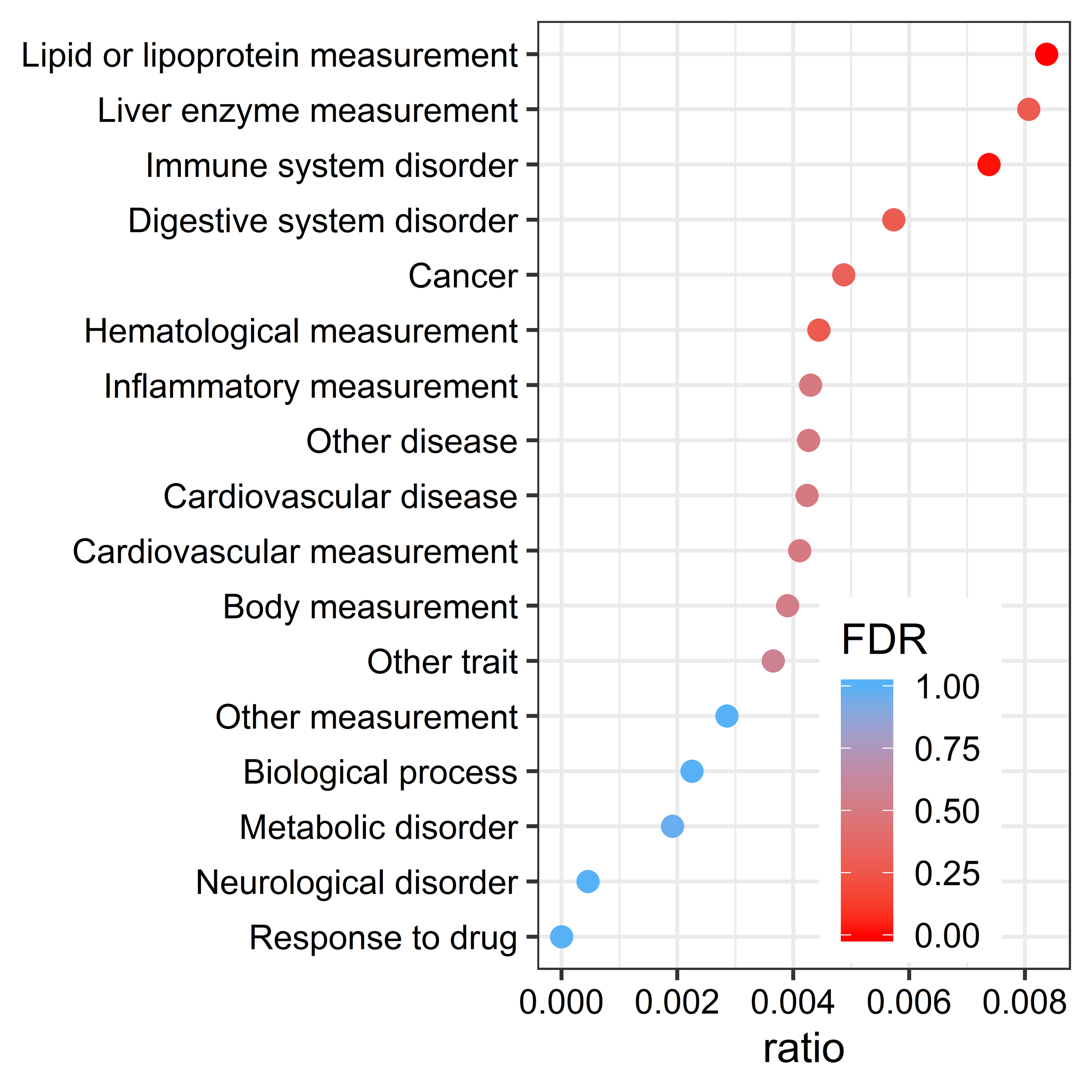

Supplement: S10 Fig — We also used 1000 Genomes YRI population to prune GWAS variants and find GWAS tagging variants (related to Fig 3B). The corresponding GWAS were significantly enriched in the following ontologies: Lipid or lipoprotein measurement FDR-corrected p value = 4.98e-07, Immune system disorder FDR-corrected p value: 1.75e-02. (TIF) [file pgen.1008662.s010.tif]

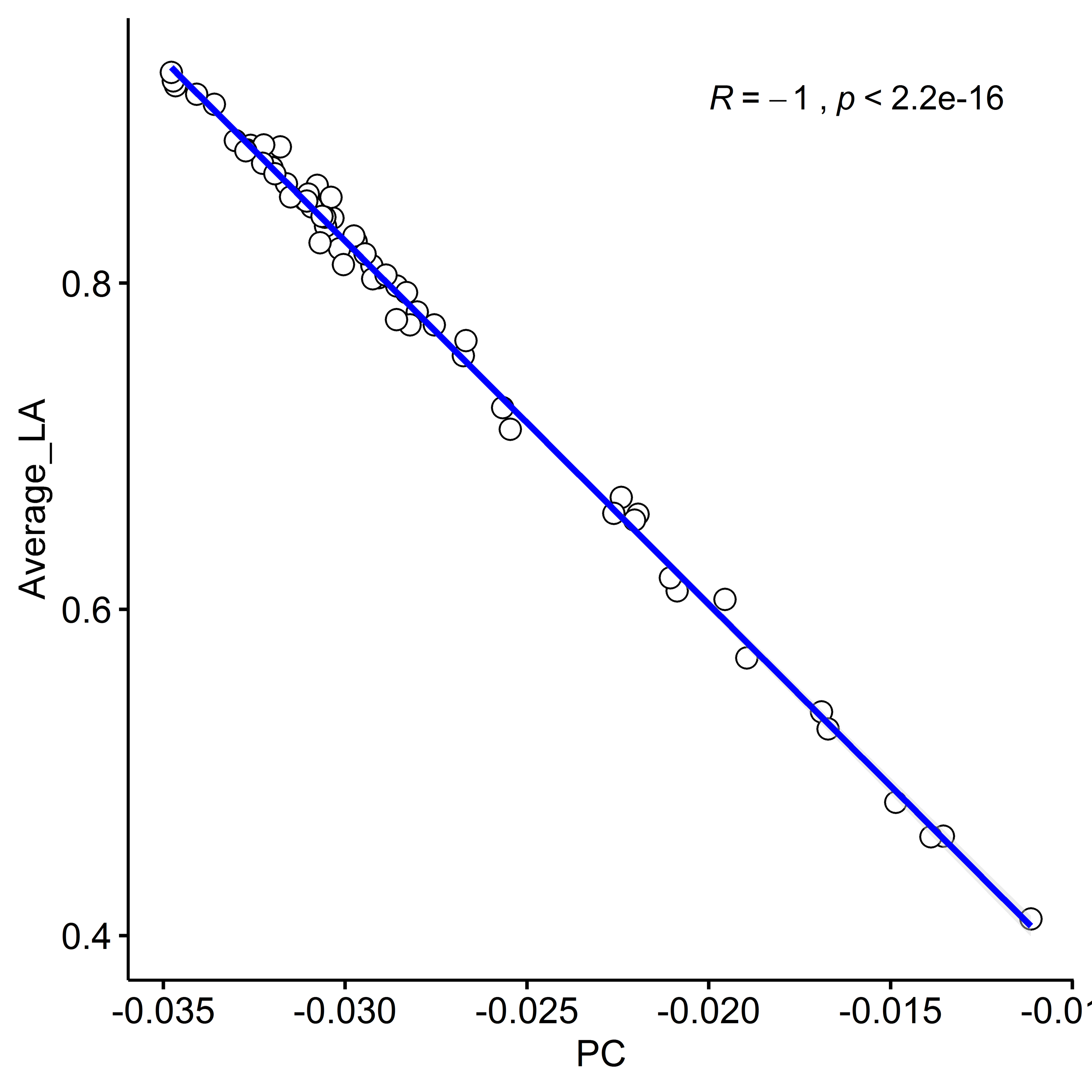

Supplement: S11 Fig — The LA ancestry was estimated with RFMix and averaged across the genome. Here we showed a high correlation between the averaged LA ancestry and the first PC in AA hepatocyte data. (TIF) [file pgen.1008662.s011.tif]

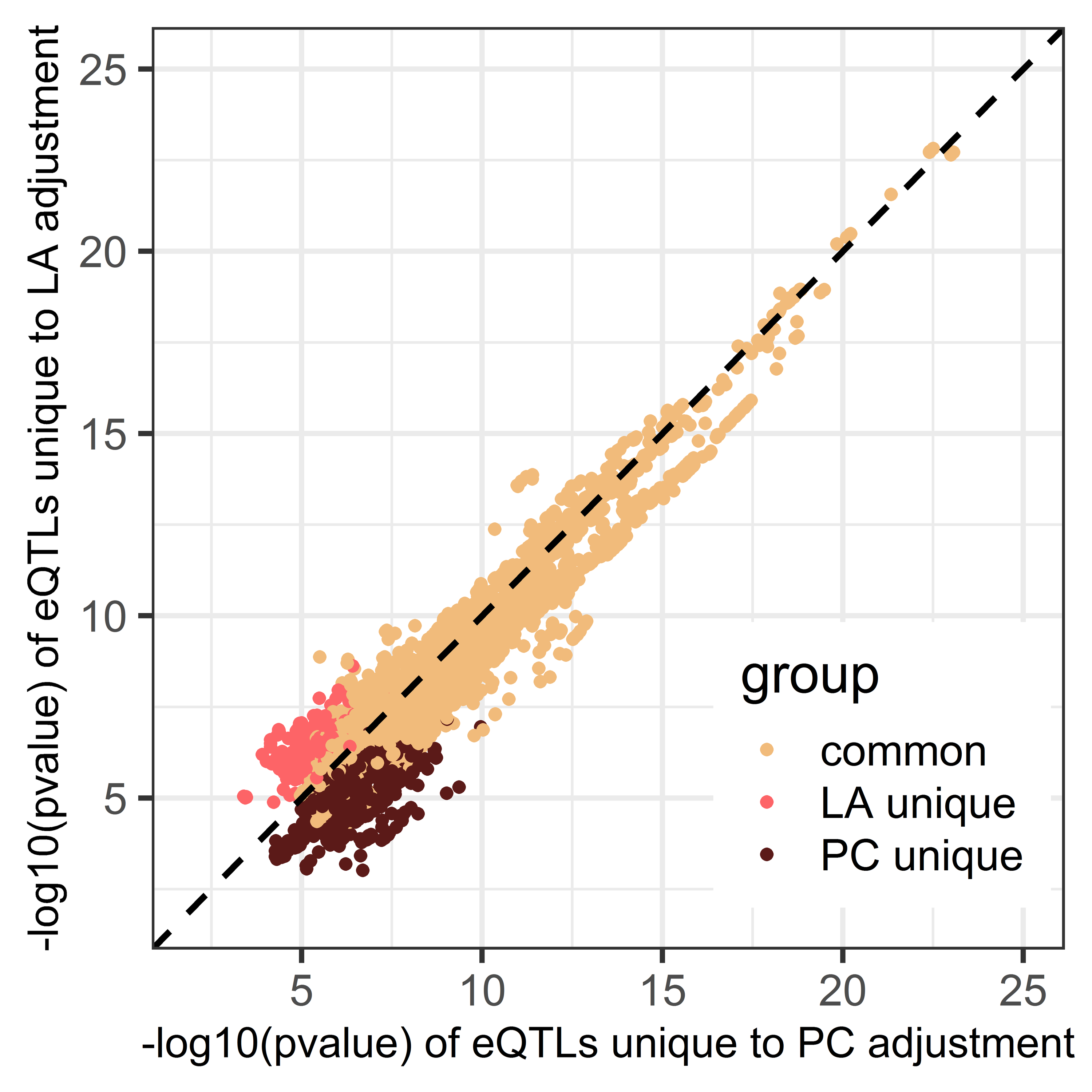

Supplement: S12 Fig — We mapped eQTLs using LAMatrix which adjusts for local ancestry instead of PC in the eQTL mapping for AAs. We identified 1,179 additional eQTLs representing potential novel eQTLs found by accounting for local ancestry structure. (TIF) [file pgen.1008662.s012.tif]

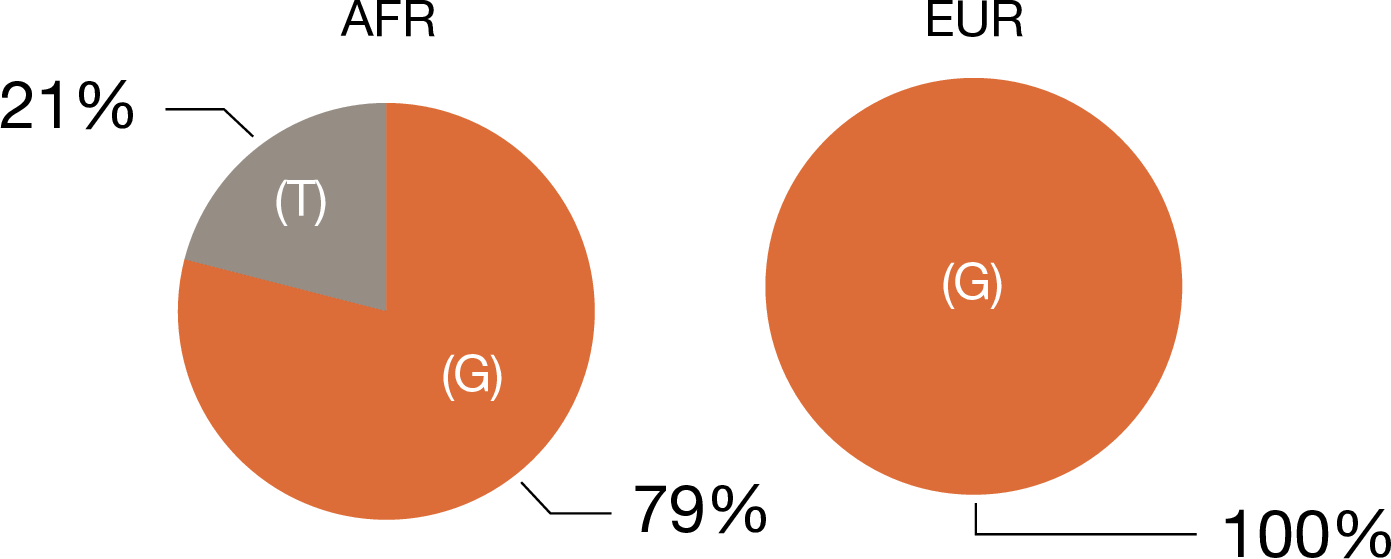

Supplement: S13 Fig — (TIF) [file pgen.1008662.s013.tif]

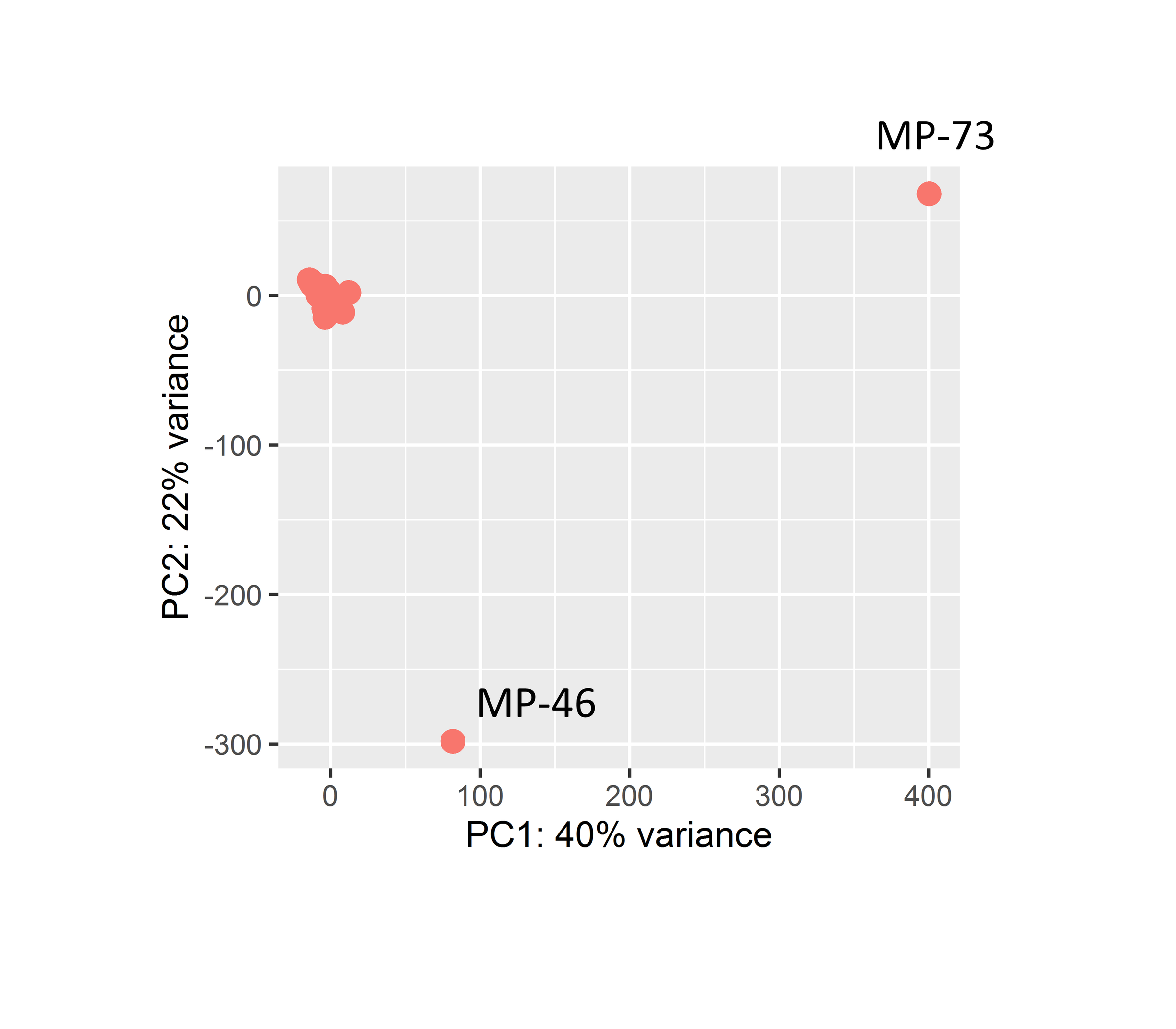

Supplement: S14 Fig — Gene counts were normalized using regularized log transformation and principal component analysis (PCA) was performed with DESeq2. PC1 and PC2 were plotted to visualize sample clustering. (TIF) [file pgen.1008662.s014.tif]
