# Supplementary material for: Discovery of novel hepatocyte eQTLs in African Americans
Source: PLoS Genet. 2020 Apr 20;16(4):e1008662. doi: 10.1371/journal.pgen.1008662 (PMC7192504; doi:10.1371/journal.pgen.1008662)
Supplement: Attachment — Submitted filename: response_letter.docx [file pgen.1008662.s015.docx]

Please find our responses below to all comments from reviewers.  Reviewer comments are in *italics* followed by authors’ response.

*Reviewer 1:  Overall, I feel that the analyses in the paper are structured logically and that the analyses provide convincing evidence for the existence of population-specific eQTLs in AAs. Despite the sample size of n = 60, which some might argue is a bit small, the authors show fairly convincing evidence of genetic variants with obvious allelic effects on expression. I appreciate the authors’ efforts to increase the population diversity of study subjects in eQTL mapping studies and feel that they have made an important contribution to the eQTL literature.*

We are encouraged that all the reviewers see the importance of this work and the contribution it makes to the eQTL literature.

*1. In general, the paper would benefit from meticulous copyediting. There are several instances of grammatical errors or strange phrasing, particularly with articles (“a”, “an”, and “the”). See, e.g. lines 87 (“The eQTL mapping…” vs. “eQTL mapping”) or line 115 (“previous GWAS findings at SORT1 gene” should be “… at the SORT1 gene.”).*

The paper has been thoroughly proofed and all grammatical errors have been corrected.

*2. Normally, the subsection headers for results use titles that explain the scientific question to be answered in that section. Some of the headers, particularly lines 119, 230, and lines 246, state what was done but not what was learned.*

We have updated the headers of each section to be more informative of the analyses and what was learned.

As such, line 119 (now line 155) has been changed to “Discovery of unique eGenes and eQTLs in AA hepatocytes and comparison to GTEx liver eQTLs”.

Line 230 (now 279) has been changed to “Exon eQTL mapping uncovers potential splicing variants”.

*3. The authors often state either explicitly (line 76) or implicitly (lines 93, 161, and Fig 3D) that AAs are an African population. While AAs are considered part of the 1000 Genomes AFR superpopulation, they are not exactly African: AAs are an admixed population with high average levels of African ancestry. Therefore, on line 93, where the authors state that “gene expression in Africans [is] unexplored.”, I encourage the authors to consider if statements like this in the paper are perhaps better phrased as something like “gene expression in populations with high levels of African ancestry,” akin to the language that appears in the manuscript cover letter.*

Thank you for this comment and we whole-heartedly agree. The sentence has been changed to better reflect the fact of African Americans as an admixed population (see line 98).  We have also changed Figure 4D to represent the LD structure of ASW instead of YRI.

*4. Expanding on comment (2), the authors use LD maps from 1000 Genomes CEU and YRI populations in Fig 3D. As I understand it, the point is to argue that the LD structure in Africans differs from Europeans, and that this observation matters for the resolution at which eQTLs can be mapped in AAs. In that vein, it is curious that the authors did not use the 1000 Genomes ASW (African-Americans from the Southwest United States), since their study cohort is genetically closer to ASW than to either CEU or YRI. ASW could therefore provide a more comparable LD structure for the authors’ sample.*

Thanks for this comment. We have changed Figure 3D (now Figure 4D) to represent the LD structure of ASW instead of YRI.

5. *Line 107: The authors state that AAs have a large portion of AFR contribution to their genomes and a smaller EUR contribution. This is certainly intuitive to anybody who knows the history of AAs, but it is also an empirical observation of the genetics of AA individuals which the authors should justify. One paper that does this nicely is Baharian et al. (DOI: 10.1371/journal.pgen.1006059)*

Thank you for the suggestion.  We have added this citation to the manuscript in line 114.

*6. Line 110: The “first eQTL mapping in AA hepatocytes” is potentially contentious, since a casual reader may ask about the 15 AAs in the GTEx eQTL map of liver tissue. Presumably the human liver is not solely composed of hepatocytes, but this nuance is not mentioned here; instead, it appears in the Discussion on line 310. If there is particular utility in clean hepatocyte expression profiles uncontaminated with other liver cells, then the authors should state this in the Introduction and repeat it in the Discussion. In the meantime, it sounds more accurate for the authors to state that theirs is the “largest eQTL mapping to date in hepatocytes derived exclusively from AAs” or something similar. Adding the sample size (n = 60) at this juncture would help, along with a reason why the sample size may feel small when compared to the gargantuan modern GWAS sample sizes (e.g. deriving human hepatocytes requires actual human livers, which one can easily harvest in model organisms but not in human).*

The introduction now reads at line 106 “The Genotype-Tissue Expression Project (GTEx) Consortium has added to our understanding of the eQTL landscape using a multiethnic cohort. However, even in this large effort, only 15 individuals of African ancestry were included in the liver eQTL mapping (26), making extrapolation from this cohort under-powered. Moreover, GTEx eQTLs were mapped in liver tissue and hence represent the transcriptome of multiple cell types.”

Additionally, Line 117 now reads “Here, we have performed the largest eQTL mapping to date in hepatocytes derived exclusively from AAs (n = 60).  We identified AA-specific eQTLs (found only in our AA dataset) and overlapping eQTLs (those found in both datasets) by comparing our results to those from the GTEx liver dataset.  While the sample size for our study remains smaller than many GWAS efforts to date, human hepatocytes require human liver samples which are difficult to obtain, with AA donor livers being rarer still”

*7. Line 127: I encourage the authors to cite a reference to KEGG here. There are numerous KEGG papers that fit the bill, but it should suffice to cite Kanehisa et al. 2016 (DOI: 10.1093/nar/gkv1070)*

Due to the change of the focus to LA-based eQTL results, the eGenes are no longer enriched in KEGG pathways and we have removed this result from the paper.

*8. Line 134: Presumably a “lead eQTL” is the eQTL of largest effect for an eGene. The authors should clearly define what “lead eQTL” means here.*

We have added the definition of lead eQTL as follows, “Of the 210 overlapping eGenes, only 25 share the same lead eQTL (the most significant eQTL for each gene)...” in line 162.

*9. Fig 1E: the panel for TMEM116 expression shows that GTEx has only two samples with an allele dosage of 1 at the identified eQTL. The authors note this on lines 161-162 and assert that the SNP “is unique to African population[s]” and has a low MAF in GTEx. I have two minor qualms here. Firstly, the authors make an assertion without justification, which they can easily rectify by providing a (supplementary) figure showing the MAF of that variant in, say, all 1000 Genomes populations, akin to what Ensembl generates for variant lookups. Secondly, in Fig 1E, box-and-whisker plot for two data points is uninformative since the two points merely demarcate the extremes of the whiskers. The authors should consider if it is not more accurate to simply plot the dots for that case.*

We have now added the allele frequency of the exemplar SNP to a supplementary figure (S13 Fig).  While the reviewer is correct to note that with only a few points in the box and whisker plot and the style of the plot is not particularly informative for this data, for the aesthetic of the figure (as the other examples do benefit from this type of visualization), we have kept this figure as is.

*10. Fig 2: Why did the authors present panels 2A and 2B as box-and-whisker plots, but panels 2C and 2D as bar plot with error bars? It seems like the box-and-whisker plot is more informative here. Note that a similar question could be raised about the panels in S9 Fig. But S9 Fig shows adjacent bars with multiple (3+) colors, and a box-and-whisker plot with 3+ adjacent colors is probably harder to interpret than the current S9 Fig.*

In Figure 2C and 2D (now 3B and 3C), we plotted the estimates of odds ratios.  We have now graphed them (Figure 2B, 3B, 3C, S9 Fig) as forest plots to better represent the mean and 95% confidence intervals.  We have removed Figure 2B per comment 30.

*11. Fig 2 and Fig 3B: The bars and box-and-whisker plots presented here would be more complete if the authors added the data points underlying the summaries, as they did in Fig 1E and Fig 5A.*

Figure 2A (now 3A) represented a total of more than 37k points, hence we have now included violin plots to show the underlying distribution.

Figure 3B (now 4B) represented the enrichment of GWAS EFO categories that intersect with AA hepatocyte eQTLs, thus the number of points that underlies this would not be informative as it is a ratio.

*12. Lines 224-225: The authors speculate that the higher number of trait associations from overlapping eQTLs (those in GTEx and their AA cohort) vs. the AA-specific eQTLs could be due to “the biased representation of European cohorts in the GWAS catalog.” Could this not also occur with larger GWAS sample sizes? Is it known if genetic markers identified by GWAS in AA cohorts are more or less likely to associate with the AA-specific eQTLs? The authors’ assertion here would benefit from a more detailed explanation.*

Only 336 AA GWAS (in which only AAs are used in the discovery cohort of the GWAS) are reported in the GWAS catalog (2019-03-22 version) out of a total of 85,851. This makes the analysis suggested difficult as the number of studies is quite limited and non-overlap of AA specific SNPs to AA GWAS may merely reflect the limited phenotypes considered in comparison to the whole catalog.  To address the contribution of larger GWAS size improving the power the detect overlap with mapped eQTLs, we have modified the following sentence in line 262, “The greater proportion of traits associated with overlapping eQTLs may be due to the biased representation of European cohorts in the GWAS catalog as well the growing number of GWAS with large sample sizes (20)”

*13. Fig 4: Is it possible to make the lines in these two panels color-blind friendly? They are already somewhat tricky to distinguish, and probably more so for readers with deuteranopia.*

Thank you for this important comment. We have replotted the Figure 4 (now Figure 5) with different colors that should be more readable to color-blind readers.

*14. Line 251: Is there a particular reason for the choice of the FDR threshold 0.1 here? In other places in the paper (lines 409, 452, 674, 735) I see that the authors use an FDR threshold of 0.05. Do the results of the local ancestry eQTL mapping change for FDR-corrected p-values < 0.05?*

We now added the results of using FDR < 0.05 to identify significant associations of LA to gene expression in line 144.  “We found 137 genes whose expression was associated with local ancestry at FDR < 0.1 and 65 genes at FDR < 0.05”. All eQTL mappings (both PC adjusted and LA adjusted) are reported at the FDR threshold of 0.05.

*15. Lines 200, 228, and 307: Following up on Comment (13), I am confused by these statements about FDR. FDR is normally stated as an adjustment procedure with an alpha level against which to compare adjusted p-values. Line 200 does not state what the FDR threshold is. Line 228 contains two statements of “FDR = x“, where I think the authors mean “FDR-corrected p-value = x”; again, the FDR threshold is not stated here. Line 307 clearly states “FDR = 0.143” which is an unusually high FDR threshold, and which I think is also referring to an FDR-adjusted p-value; a p-value at 0.143 is certainly not significant at FDR < 0.05 or 0.1, for example, and explains the authors’ statement about rs9923231.*

The reviewer is correct. This line in 336 now reads, “Although this SNP is a significant eQTL in GTEx, its association in the AA hepatocyte data does not rise above FDR threshold (FDR-corrected p-value = 0.23)”  All other instances of this have also been corrected (see line 233 and 267).

*16. Lines 291-292: the authors state, “It is possible that eQTLs related to drug response are discoverable only under specific contexts” as a plausible explanation for why so few eQTLs appear ontologically related to drug response. But a simpler possibility is that most researchers simply don’t have widespread access to well-designed eQTL studies of drug response. This is an important gap in the scientific literature, and the authors have license to remind readers about the pressing need for these kinds of studies.*

Thank you for this suggestion. We have revised this paragraph to read, “Additionally, drug response GWAS (n=541) represent 0.63% of the overall GWAS catalog with many of the significant findings representing coding variants, which may not change gene expression but change the functional activity of the protein (49).” in line 330.

*17. Lines 303-304: The authors state that the sample size (n = 60) is a limitation of the study, and “type I and type II error may exists” [sic]. This is an odd choice of words, and perhaps a bit unfair to the authors, since the possibility of false positives and false negatives will exist so long as the FDR threshold is greater than 0! Certainly, a greater AA sample size may discover more AA-specific eQTLs, or it could also replicate many eQTLs already in GTEx as well but with a smaller effect size in AAs. No rational geneticist would argue that this study paints a complete picture of the genetic architecture of gene expression in AAs. Perhaps the authors can rephrase this statement as an admission of the limited statistical power available in their study, and that the interpretability of their results should be tempered accordingly. At the same time, the authors have demonstrated markers that show clear allelic effects on expression, so I feel that they should not sell themselves short here.*

We are encouraged by the reviewer’s understanding of this area of genomics.  We whole-heartedly agree. Much of the work in minority population is maligned for small sample size with little acknowledgment of the gapping lack of data in these populations and additions (even of a small size) to help to elucidate population specific effects.  We have now changed this sentence to read, “Our study has several limitations. Firstly, the sample size of our AA cohort is relatively small, and thus limited the power to discover AA-specific eQTLs with small to moderate effect sizes.” in line 333.

*18. Lines 307-309: The authors point out that GTEx harvested postmortem tissue, and that this may introduce heterogeneity when comparing against their hepatocyte cell lines. There is some literature on how gene expression from postmortem tissue differs from that in living tissue samples, which I encourage the authors to discuss and cite; see, e.g. Ferreira et al. 2018 (DOI 10.1038/s41467-017-02772-x), Tolbert et al. 2018 (DOI: 10.1016/j.gene.2018.06.090), and Zhu et al. 2017 (DOI: 10.1038/s41598-017-05882-0) However, in Line 324, the authors state that their hepatocyte cell lines derive from non-transplantable organs. Since humans cannot survive without a liver, it seems reasonable to speculate that these hepatocytes could derive from deceased donors. Is this the case? Can the non-transplantable nature of the organ also affect gene expression? If not, then the authors should clearly state so, and justify how their hepatocyte cell lines are indeed different from postmortem cell lines.*

We agree with the reviewer that factors such as postmortem interval (PMI) do affect gene expression in post mortem tissue samples and we have added this into the discussion.  But we would like to note that the effect of PMI on living hepatocytes is less clear. Most of the work on the effect of PMI on gene expression has been conducted on flash frozen tissue which does not contain living cells whose expression can still be modulated (as is the case with primary hepatocytes).

We obtained our hepatocytes from multiple sources due to the relative rarity of African American livers.  Some were commercially obtained while some were extracted from non-transplantable livers. These details can be found in the methods.  We have performed batch and confounding correction to ensure that our samples have comparable gene expression values. The issue with the term “non-transplatable” is that this does not always indicate a disease condition with the donor organ. There are instances when the issue of transplantability were with the recipient and hence the organ did not go for transplant.  Therefore, it is complicated to generalize about the health or non-health of the livers used in this study. We excluded livers from those with active cancer or a history of hepatocarcinoma. These details have been added to the methods to clarify how the samples were obtained.

*19. Lines 366-368: The authors state that they discarded three samples based on PC plots of gene expression. I encourage the authors to add these plots as a supplementary figure, along with one that shows what constitutes a “normal” gene expression pattern in their sample.*

We have added the PC plot as requested as S14 Fig.  We want to clarify that one sample was excluded due to low African ancestry and two samples were excluded due to the PCA plot of gene expression instead of three.  This section now states “Two samples with distinct expression patterns were excluded as outliers.” in line 406. We have also updated the flow chart (Figure 1A) to correctly reflect these exclusions.

*20. Line 400: “… 6,453,712 SNPs” is more readable than the same number with no comma delimiters.*

We have changed this to 6,453,712 as suggested in line 459.

*21. Line 415: If the authors refer to centimorgans here, then I think that “cm” should be “cM”.*

We thank the reviewer for pointing out this typo. We have made the change in line 485.

*22. References: Reference #37 is the preprint version of Reference #15. Reference #54 is a repeat of Reference #52.*

We have removed the duplicated references and checked the references thoroughly.

*23. Line 768, Fig 3 caption: in fairness to the EBI, which is a co-sponsor of the GWAS Catalog, perhaps the authors should write the “NHGRI/EBI GWAS Catalog”.*

We have changed the text to NHGRI/EBI GWAS Catalog in line 501, 895, 897.

*24. S2 Fig: The covariate names appear on the diagonal of this multipanel plot. This text on the diagonal is unnecessary since those covariate names appear as column and row names. Furthermore, the text is hard to read. I suggest that the authors simply remove it.*

We have removed the text in the diagonal of S2 Fig.

2*5. S6 Fig: It is confusing to have AA-specific eQTLs described in the context of the “AA” label in this figure. At first glance, I thought that AA-specific eQTLs all had higher average MAF than GTEx eQTLs, whereas the intent of the figure (I think) is to show that eQTLs estimated in the authors’ AA study cohort and replicated in GTEx have low MAF in GTEx. I encourage the authors to consider clarifying the labeling in this plot.*

We have changed the legend to be “MAF in AA” and “MAF in GTEx” in S6 Fig to be clear that it is showing the MAF in two different datasets for the same set of eQTLs.  We would like to point out that these are the AA-specific eQTLs (i.e. they were not significant eQTLs in GTEx analysis) and the distribution shows that these SNPs have low MAF in GTEx as compared to our AA cohort and hence may have been under-powered in the GTEx analysis.

*Reviewer 2:  This is an important study to publish given the relative paucity of gene expression data in non-European and minority populations, and that liver QTL studies are particularly important for refining complex disease associations. Some of the findings of the paper underscores this importance (although I do outline below where I think some conclusions may be inappropriate). In general (with some exceptions) the authors seemed to have used robust data analysis pipelines, did appropriate statistical analyses, and generated good quality data. However, I do think some major revisions are necessary for publication.*

We thank the reviewer for highlighting the importance of our study.

26. *According to this now somewhat outdated review paper,* [*https://www.ncbi.nlm.nih.gov/pmc/articles/PMC3418580/#B17*](https://urldefense.proofpoint.com/v2/url?u=https-3A__www.ncbi.nlm.nih.gov_pmc_articles_PMC3418580_-23B17&d=DwMGaQ&c=yHlS04HhBraes5BQ9ueu5zKhE7rtNXt_d012z2PA6ws&r=Z82D5BofkW8K7bpdtaj3QROGd7-VymHDTObrhY9bEt2bDfQZB4GHiEW9OUotWV2B&m=AYG2ltWmyjlMkVCMpvoOwFeiYPx4_BcdHHc9m0e3iT8&s=dpPz7D3yA7E1Sd7Uxytv6QHAq7gR-oQxqIlrAYgmPnk&e=)*, there are other eQTL liver studies, one of which includes African American subjects. Did the authors consider looking for resources outside of GTEx to compare their liver eQTLs with? Do they have a good rationale for only comparing their data only to GTEx, e.g. availability of RNASeq data, proper study design, etc.?*

We thank the reviewer for the opportunity to clarify our choice of replication cohort as this is an on-going question for study like ours.  The Innocenti et al. cohort referenced above is a valuable resource, but because gene expression was quantified through microarray in this study, comparison to our data would be less than ideal.  GTEx used RNA seq quantification and hence is gene expression quantification is more comparable. If our eQTLs were not to overlap with the eQTLs in the Innocenti dataset, in addition to population-specific factors and hepatocyte versus liver tissue differences as possible explanations, dynamic range of quantification would also have to be considered.  We have added a sentence within the discussion in line 303 to address this issue.

“While other eQTL studies have AA samples in their analysis (47), the number of samples has been much smaller and gene expression was quantified via gene expression microarrays.”

*27. The GTEx sample size is ~ 3 folds larger than the new AA hepatocyte sample set. If the authors account for this sample size difference (by e.g. randomly selecting the same number of subjects from GTEx to identify eGenes and eQTLs, and repeating this a few times), how does the number of overlapping vs unique eGenes and eQTLs between the 2 data sets compare then? I.e. can you discover more with African ancestry eQTLs than European ancestry eQTLs if all else is equal? (Although point 4 below may make this somewhat difficult to compare directly)*

We have conducted the analysis suggested by the reviewer and as expected the number of eGenes found in subsampled GTEx drops dramatically to 616 (average over 10 iterations, S.D.=36.8).  Of these only 108 (S.D.=5.3) now overlap with those found in the AA cohort (leaving 169 as AA-specific). This suggests that, with all else equal, there are eQTLs and eGenes to be found using African ancestry individuals to map eQTLs.  It would also suggest that GTEx required larger samples size to detect eQTLs we can detect in our smaller AA cohort. While this is interesting, we cannot distinguish if these changes are due to population specificity or tissues heterogeneity, therefore we have not drawn any new conclusions from these results. We are open to the addition of this data to the paper if the reviewers/editors find the results necessary to the overall message of the paper.

*28. On lines 204-229 the authors argue for increased fine-mapping utility because less AA hepatocyte eQTLs overlap with GWAS variants. The authors argue that this is due to decreased LD, but is it not also likely to be due to the smaller sample size of the AA hepatocyte data set? I suggest using the same framework as in 2 above to tease out the effect of sample size on the result set.*

We thank the reviewer for pointing out the sample size difference. We performed comparison between subsampled GTEx and AA hepatocytes and added the following to the results in line 251.

“To exclude sample size differences, we randomly sub-sampled the GTEx European liver dataset to match the sample size of AA hepatocyte data and performed eQTL mapping.  The sub- sampled GTEx eQTLs averaged 19 eQTLs (S.D. = 3.2) for SORT1 across 10 iterations. We performed fine-mapping with CAVIAR with the sub-sampled GTEx European and AA hepatocyte eQTL mapping results and found CAVIAR identified five eQTLs in the sub-sampled GTEx European cohort and only two eQTLs in the AA cohort in the 95% credible set.”

29. *A major limitation to the conclusions of the study is the difference in liver tissue type in GTEx (noted by the authors in the Discussion, but perhaps not strongly enough). With the absence of additional supporting data, e.g. showing overall comparable gene expression patterns in GTEx vs AA. hepatocytes particularly for eGenes unique to AA hepatocytes, one cannot make strong claims about the additional discoveries being due to the African ancestry of the hepatocyte sample set (I don't think a higher MAF spectrum of the new eQTLs discovered is conclusive on its own). Either such additional supporting data should be added, or especially the abstract conclusions and author summary should be less strongly stated.*

Thank you for this insightful comment.  We agree that our results may represent either AA specific findings or hepatocyte-specific findings.  We have changed both the author’s summary and the discussion to make this point clear.

In summary line 63, “Lastly, we show that by identifying both population-specific and hepatocyte-specific eQTLs in African Americans, we can identify potential genes that drive disease.”

In discussion line 345, “Therefore, eQTLs found in our AA cohort may represent either AA-specific eQTLs or hepatocyte-specific eQTLs.  As noted in the results, only 8 eGenes were found in the AA hepatocyte dataset, which were not expressed in the GTEx liver dataset.”

*30. Lines 413-418: LDSC does not account for the long-range linkage disequilibrium patterns in admixed populations such as African Americans and these results can therefore be misleading. Either the results should be removed from the paper (as I don't think it is integral to the paper) or the analysis should be repeated using a more appropriate method e.g.* [https://www.biorxiv.org/content/biorxiv/early/2018/12/20/503144.full.pdf](https://urldefense.proofpoint.com/v2/url?u=https-3A__www.biorxiv.org_content_biorxiv_early_2018_12_20_503144.full.pdf&d=DwMGaQ&c=yHlS04HhBraes5BQ9ueu5zKhE7rtNXt_d012z2PA6ws&r=Z82D5BofkW8K7bpdtaj3QROGd7-VymHDTObrhY9bEt2bDfQZB4GHiEW9OUotWV2B&m=AYG2ltWmyjlMkVCMpvoOwFeiYPx4_BcdHHc9m0e3iT8&s=jYhayslQHAvnvnem1wVQcfJwshYMl9ERI-Kf5NdSjPk&e=).

Thank the reviewer for the comment. We have removed the analysis from the paper.

*31. - Line 50: Preferably do not use the word biomarkers, as strong biomarkers for disease was not discovered by this study, and this may mislead people who read the author summary*

We have changed the genomic biomarkers to genetic variants in line 53.

32. - *Line 57-58: Sentence does not make sense: "This is an important as fine-mapping of causative variants has relied heavily on function validation."*

The sentence has been changed to in line 60 “We significantly decreased the number of potential causal variants at GWAS loci that overlap with our eQTLs.  This is important as fine-mapping of causal variants has primarily relied on functional validation.”

*33. - Line 60: "gene drivers" - suggest rephrasing as "genes that drive disease"*

This has been changed as suggested in line 64.

*34. - Line 76: "population" should not be singular*

We have changed to populations in line 79.

35. - *The authors should very briefly define what an eGene is when first used, for readers who are not familiar with the term*

We have added the eGene definition in line 137 “The number of PEER variables was chosen to maximize the number of discovered eGenes (genes with at least one eQTL) as previously described (S1 Fig).”

*36. - Lines 87-89 and elsewhere: "the" eQTL mapping: suggest dropping "the"*

We have removed the “the” before eQTL mapping throughout the paper.

*37. - Line 97: admixed populations (AAs)- should be admixed populations such as African Americans (AA)?*

We have changed the text as suggested in line 103.

*38. - Line 98: "for many" should be "of many"*

We have changed the text as suggested in line 104.

*39. - Line 106-107; "coming from" - suggest rephrasing "inherited from"?; suggest dropping "parental"*

We have changed to “inherited from” and removed the word “parental” in line 114.

*40. - Line 107: "This results" implies that it is admixture that is causing shorter blocks of LD in African Americans, where it is really the African ancestry that leads to shorter LD (whereas more genetic variation and different allele frequencies is a function of both African ancestry and admixture) - rephrase*

This sentence now reads, “As such, AAs have shorter stretches of linkage disequilibrium and greater amount of genetic variation with differing allele frequencies, …” in line 114.

*41. - Line 115: suggest prefacing with "the" SORT1 gene*

We have changed the text as suggested.

*42. - Fig S8: The distributions on the plot look very similar. Perhaps use log scale or violin plots to more clearly show the differences.*

We have changed this to a violin plot as suggested.

*43. - Line 173: "eQTLs groups" should be "eQTL group"*

We have removed the LD score analysis per comment #30.

44. - Line 207: "Eight-one"?

We thank the reviewer for pointing out this typo. These data have been changed due to other revision requests.  The sentence now reads, “41 GWAS variants had only one SNP in LD at the significant loci after intersecting with AA hepatocyte eQTLs.”

*45. - Line 218: "sufficient" should be "sufficiently"*

We have changed the text as suggested in line 251.

*46. - Line 219: "increase" should be "increased"*

We have changed the text as suggested in line 257.

*47. - Line 221: "potential" should be "potentially"*

We have changed the text as suggested in line 259.

*48. - Line 260: "heterogeneities" - as far as I know, heterogeneity does not have a plural form (but I may be wrong)*

We have changed the text to heterogeneity in line 297.

*49. - Line 262: and additionally, less than 5% of subjects are non-European or non-Asian (*[*https://www.nature.com/news/genomics-is-failing-on-diversity-1.20759*](https://urldefense.proofpoint.com/v2/url?u=https-3A__www.nature.com_news_genomics-2Dis-2Dfailing-2Don-2Ddiversity-2D1.20759&d=DwMGaQ&c=yHlS04HhBraes5BQ9ueu5zKhE7rtNXt_d012z2PA6ws&r=Z82D5BofkW8K7bpdtaj3QROGd7-VymHDTObrhY9bEt2bDfQZB4GHiEW9OUotWV2B&m=AYG2ltWmyjlMkVCMpvoOwFeiYPx4_BcdHHc9m0e3iT8&s=ZQskzUAGJqFXEiUATrlw_kdSbXO2PEwGqsAr3ve61dA&e=)*)*

We have added the suggested text and reference in line 299.

*50. - Lines 293-302: This was not described at all in results - move to results and briefly put in discussion?*

We have moved the section to results to line 269-278.

*51. - Line 340: but you would have no missing data with imputed data so why filter on missingness?*

We apologize; this was a typo. Missingness was only considered at the pre-imputation stage.  We have changed the text to accurately reflect our QC process.

*52. - Line 342: "the gene dosage" should be "gene dosages"*

We have changed the text as suggested in line 381.

*53. - Line 398: According to* [*https://gtexportal.org/home/tissueSummaryPage*](https://urldefense.proofpoint.com/v2/url?u=https-3A__gtexportal.org_home_tissueSummaryPage&d=DwMGaQ&c=yHlS04HhBraes5BQ9ueu5zKhE7rtNXt_d012z2PA6ws&r=Z82D5BofkW8K7bpdtaj3QROGd7-VymHDTObrhY9bEt2bDfQZB4GHiEW9OUotWV2B&m=AYG2ltWmyjlMkVCMpvoOwFeiYPx4_BcdHHc9m0e3iT8&s=zHUTIolahZUY5Vqp7L_j_MgTh7nSXlUZmDBCDVBhvps&e=)*, there are 208 liver samples with genotype data in GTEx - why the discrepancy in numbers?*

At the time of analysis, we compared our results with GTEx v7 (which was the most recent version available at the time). Since that time, GTEx has release v8 data.  The version of GTEx used in our analysis is included in Methods in line 456.

*54. - Line 404-405: This implies that opposite effect direction eQTLs were labelled AA-specific eQTLs - surely not?*

We thank the reviewer for the opportunity to clarify.  We considered overlapping eQTLs are SNPs at the same position with concordant direction of effect.  AA-specific eQTLs are eQTLs not found in GTEx. If a SNP is found in GTEx and has a discordant effect, it would not be considered as an AA-specific eQTL (as it was found in GTEx).  In our analysis only one SNP met this description. In other words the SNP-gene pairs labeled as AA-specific are unique and not found in GTEx.

We have changed the method in line 462 to make it clear.

“AA hepatocyte eQTLs that were also significant in the GTEx dataset and have concordant direction of effect were identified as overlapping eQTLs.  AA hepatocyte eQTLs that were not found in GTEx were identified as AA-specific eQTLs.”

*55. - I suggest that at the very least, supporting Information files which report all significant eQTLs, exonQTLs, local ancestry QTLs for use in fine-mapping of other studies be   provided (so researches don't have to create these data from scratch from dbGAP and GEO), and possibly even all summary statistics*

We thank the reviewer for the comments. We have uploaded the results (local ancestry adjusted eQTL mapping, PC-adjusted eQTL mapping, and exon eQTL mapping) to figshare.

We have added the link to these data to the paper in line 140.

 “Summary statistics for this analysis are publicly available through Figshare (https://figshare.com/projects/AA_hepatocyte_eQTLs/72635).”

*56. Throughout the manuscript the authors report the total number of significant expression-SNP associations (eQTLs) which is the result of a marginal association scan. This procedure is standard, however it is misleading to only report the total number and not the estimated independent number of eQTLs. For example, at a given gene if a causal eQTL exists (either directly in the data, or through tagging), then any other nearby SNP that is in LD should also exhibit a significant marginal effect (barring power to detect in the first place). I think it’s fine to report the total number of eQTLs found, but the number of independent signals should be emphasized as that is more descriptive of the underlying biology.*

*To remedy this, I encourage the authors to perform a conditional analysis on the lead eQTL to estimate residual regulatory action conditioned on the lead hit. This, coupled with the lead eQTLs, would paint a much clearer picture of the regulatory landscape for hepatocytes in AA individuals. As such, all downstream comparative analysis with EUR/GWAS catalog should prioritize using the lead and secondary SNPs over the complete set of associated SNPs.*

*This is made apparent when the authors report 236 eQTLs for SDHC and possible link to hepatocellular carcinoma. It is highly unlikely that this gene has 236 SNPs involved in regulation, compared to the more likely 1-2. If either the lead (or secondary eQTL if it exists) are not rs3935401 then the authors should remove this statement.*

In addition to the eQTL and eGene numbers which give an overall sense of the proportion of genes with genetic regulation, we have performed the conditional analysis suggested by the reviewer as follows to identify independent signals for each eGene. This was added to Methods in line 448.

“We identified the most significant eQTL (lead eQTL) for each eGene and regress the effect of the lead eQTL to obtain the residual gene expression as Jansen et. Al. (75).  We performed eQTL mapping with the residual expression and SNPs within 1Mb of the gene. We adjusted p values of all cis SNPs for each gene using Benjamini and Yekutieli (BY) method and used the same BY threshold to call secondary eQTL as was done with primary eQTLs.”

And Results in line 142:

“Using conditional analysis, we identified 240 secondary eQTLs for 2 genes (*HCG4P7* and *PPIL3*, S7E Fig, S7F Fig).  Of the 240 secondary eQTLs identified, 217 were not identified in our primary analysis.”

However, we feel that the overlap using only the lead eQTL and/or independent eQTLs may underestimate the contribution of these SNPs to GWAS results.  Specifically the associations reported in the GWAS catalog are dependent on the genotyping platform used and the imputation reference genome used (if imputation was even conducted).  Hence, all possible tagging SNPs to a GWAS hit may not be represented in the catalog. Furthermore, we chose to use the overlapping method as opposed to the *coloc* method because of potential differences in LD and allele frequency between the populations used in the GWAS and the AA population used in our eQTL mapping.  By including all eQTLs mapped in our results, we lessen the effects of these factors on our analysis and provide a better representation of eQTLs which inform GWAS findings.

*57. The authors report increased Fst at AA-specific eQTL SNPs compared to EUR-shared eQTL SNPs. From a purely descriptive standpoint I think this analysis is fine for emphasizing frequency differences between populations, but the typical interpretation of Fst is a consequence of selective pressure which I think would need much more work to show. Given that, the authors should clearly state the caveats of this interpterion of Fst, and state that one reason is differential tagging, and therefore power to detect, of the ungenotyped causals between populations.*

The Fst analysis in this study was only to show that the eQTLs we labeled as AA-specific had greater allele frequency differences between populations and hence the use of African Americans in this study may have enabled their discovery.

We had added the following sentence so that the intent of this analysis is clear, “It should be noted that we did not test for selective pressure in this analysis and that Fst was only used to characterize the difference in allele frequency between populations in AA-specific versus overlapping eQTLs.” to line 207.

*58. Ascertaining on the shared and AA-specific eQTLs, the authors report LD score differentiation and how that may contribute to differentiation. Can the authors include the EUR LDscores at these same SNPs for a baseline comparison? If avg LDscore at the AA-specific eQTLs is lower than the LDscore for the same SNPs in EUR that would also support an argument regarding differential tagging of the causal eQTL.*

We have removed this analysis per comment 30.

*59. It would be interesting to perform a cross-population fine-mapping of the eQTL results for both AA and EUR to characterize the shared landscape. For example, the authors argue that the fewer number of SNPs identified in the AA data enable more precise fine-mapping compared with the EUR GTEx dataset. This argument can be made much more precise by performing statistical fine-mapping using the AA dataset compared with the EUR GTEx dataset and quantifying the expected number of causal variants at each locus. This is computed from the average posterior probability at each locus under some fine-mapping software (e.g., FINEMAP, PAINTOR, SuSie, etc).*

We have now performed the analysis suggested using a subsampling approach suggested in comment 28 to account for sample size differences.  We added the following details to methods in line 473.

“We used CAVIAR (CAusal Variants Identification in Associated Regions) (33) to statistically fine-map eQTLs in AA hepatocytes and GTEx subsampled eQTLs separately.  We applied CAVIAR to the cis eQTL mapping z scores and LD for each overlapping eGene while setting the number of causal variants to 1. CAVIAR estimates the set of SNPs that account for 95% of the posterior probability of the causal variants at each locus.”

We have also added the following to the Results in line 191.

“We performed fine-mapping of overlapping eQTLs with CAVIAR (33) for each overlapping eGene (N = 210) we estimated the casual set of SNPs underlying the eQTLs found in the AA hepatocytes analysis and the GTEx liver analysis.  To remove the effect of sample size differences between cohorts, we randomly sampled 60 samples from GTEx European cohort to match the size of the AAs. AA hepatocyte eQTLs had significantly fewer number of eQTLs in the 95% causal set as compared to the GTEx liver subsets (median AA: 15, median GTEx: 173.5, Wilcox-rank sum test: p-value < 2.2e-16).”

*60. Given that African Americans have significant admixture on average, the authors separately perform a local-ancestry adjusted QTL analysis to identify eQTLs that are driven by ancestry differences. This is well-motivated, but I do not understand why the primary analysis did not include estimates of local ancestry for each gene to begin with. This is supported by their results of stronger enrichment for H3K27ac markers in the adjusted analysis compared with the un-adjusted. The manuscript could be improved significantly if the authors reported a single set of results first adjusting for local ancestry and then separately reporting which genes associated with local ancestry estimates.*

We have now made the LA adjusted mapping the focus of the paper and added the PC adjusted results as supplementary.  We have also added the the analysis of gene expression with significant associations to LA to the results. We thank the author for encouraging us to report the work in this manner as we, too, feel it is more accurate as we outlined in our LAMatrix paper.
